# Freedom of Information (FOI) as a data collection tool for social scientists

**DOI:** 10.1371/journal.pone.0228392

**Published:** 2020-02-21

**Authors:** Joanna Clifton-Sprigg, Jonathan James, Sunčica Vujić

**Affiliations:** 1 Department of Economics, University of Bath, Bath, England, United Kingdom; 2 Department of Economics, University of Antwerp, Antwerp, Belgium; Universidad de las Palmas de Gran Canaria, SPAIN

## Abstract

This paper evaluates a method of generating a unique dataset that has been underused—a Freedom of Information (FOI) request. The UK’s FOI Act came into force in 2005, allowing the public to make requests of publicly held data. We set out to understand the determinants of the quality responses to FOI requests. We also explain how requests can be made and provide suggestions to construct effective data-driven requests. We applied for data on hate crime from all police forces and on uptake of maternity leave from all universities. We find that observable characteristics of the local area, police force and universities neither determine whether the request was fulfilled, nor the speed of response, suggesting that the data from these FOI requests are representative of the institutions from which they were requested.

## Introduction

Hate crime and maternity and shared parental leave uptake (or rather the lack of it) feature heavily in the current public discourse in Britain and are of serious concern for policy makers. The issue of hate crime surfaced at the forefront of the public debate in the UK following significant migration inflows into the country, which led to conspicuous changes in population composition [1]. Recently, however, there has been a visible surge in reported hate crimes following the country’s Referendum on the Membership of the European Union [2] [3]. At the same time, the ongoing debate around gender pay gap and attempts to close it have brought to the fore the issue of the failed implementation of the shared parental leave, which was introduced in April 2015 in the UK. Specifically, the low uptake of shared leave and low awareness of citizens of its existence have been extensively covered by media (see [4] for example).

Despite their high visibility and importance for policy-making, these topics are still heavily under-researched. This is largely due to the lack of available quantitative data, which would permit rigorous empirical (econometric) analysis. For example, while government data are being increasingly opened up and made available, they often lack variables or characteristics that would be useful or essential to the researcher. Crime datasets in the UK are one particular example of this. Even though detailed aggregate crime statistics are publicly available, the individual level crime data are not only difficult to obtain, but also difficult to match to other relevant characteristics (for a more detailed discussion, see [5]). [6], who examined the impact of tougher sentencing on crime, required crime and police data at highly localised geographical areas, which are typically not provided in published statistics. [7] exploited timing of alcohol consumption and required detailed information on the time when crimes are committed. [8] required monthly counts of hate crimes broken down by the ethnicity of a victim in their investigation of the impact of terrorist attacks on hate crime. Each of these papers used Freedom of Information (FOI) requests to gather administrative data that are not routinely available.

Similarly, the publicly available data related to uptake of maternity and shared parental leave in the UK are insufficiently detailed to permit analysis of their determinants, especially their financial conditions. This is also the case for higher educational institutions in the UK, even though they generally lead in provision of information related to any matters regarding gender equality. For example, many of the UK higher educational institutions have taken explicit steps to address gender inequality as part of the Athena Swan Charter (https://www.ecu.ac.uk/equality-charters/athena-swan/). Nonetheless, many of them do not make their policy documents publicly available. For example, often one cannot find details of their occupational maternity and shared parental leaves provided in addition to the statutory entitlements, which would allow researchers (or potential job applicants, for that matter) to gauge generosity of the employer. Similarly, many but not all universities publish their salary scales. More importantly, one may require anonymised individual level data on uptake of leave to compare and contrast the uptake at various institutions and consider key determinants. Such data are currently not available. In relation to this, recently FOI requests were used by BBC in order to write an article about the referrals of university staff to counselling services [9].

Motivated by the pressing research questions, which are also present in the current public discourse, such as hate crimes and maternity and shared parental leave uptake, and insufficient public data availability, this paper focuses on an important potential source of self-generated data that has not received much attention, nor has (yet) been fully exploited by the economists and other social scientists—Freedom of Information (FOI) requests. In line with the data gaps identified earlier, we consider two different FOI data requests and evaluate their effectiveness in 1) obtaining previously unavailable data and 2) generating representative data. In doing so we make the following two contributions to the field of economics and related social sciences; first, the data we obtained will allow us to undertake novel analyses on hate crime and family policies, which will be of academic and policy importance. Second, by demonstrating representativeness of the two independent FOI data sets we are hoping to convince researchers of their usefulness and reliability, instilling confidence in this data collection method and thus popularising its use for the future.

The FOI acts have been introduced in many countries over recent decades “in response to domestic or international pressures for transparency and good governance” [10]. The US passed an FOI act in 1966, followed by Australia, Canada and New Zealand in 1982, Ireland in 1997 and the UK in 2000 (coming into force on January 1^st^, 2005). [10] assess performance of the FOI act in the UK in comparison to other countries by considering the total number of requests made over time, percentage of requests granted and whether refusals to provide data are appealed against. FOI allows researchers access to bespoke datasets, which are typically administrative, given the FOI’s focus on the public sector, from tax records to schooling. The commitment by various governments to open up their data has led to a vast number of datasets becoming increasingly publicly available. Further, administrative data have a number of advantages over survey data. Specifically, administrative samples have universal coverage (and hence large sample sizes) and fewer issues with measurement error or attrition compared to traditional surveys [11] point out the wealth of administrative data that is collected across a wide range of domains of the public sector and how lack of access to it is threatening the dominant position of the US in economics research. At the same time countries, which allow researchers to use administrative data, have lengthy procedures in place in order to access them. FOI requests can partly help overcome this.

The aim of this paper is to understand the determinants of the quality of the responses to FOI requests. One concern with obtaining data from the FOI requests could be that those authorities that respond might be systematically different from those which do not and therefore the researcher obtains a non-representative sample. Therefore, we subsequently analyse whether there is any differential selection among institutions from which data are requested with respect to provision of the data along their observable characteristics. Specifically, we document the determinants of two particular FOI requests we made—i) for the data on hate crime from all police forces in the UK and ii) for the data on the uptake of maternity leave from all UK higher education (HE) institutions, based on their HE status as registered with the Higher Education Standards Agency (HESA). In addition, we explain how researchers can make FOI requests and provide tips on how to make those requests more successful. We do so as it is apparent that researchers are currently not fully exploiting the FOI provision to access the data, which have already been collected but are simply unpublished. [10] show that only a tiny proportion of the population make FOI requests—only one or two per thousand of the population per year. The lack of engagement with the FOI requests may be due to the fact that access to data is not always guaranteed. Although public authorities are legally obliged to respond to the FOI requests and provide data within 20 working days (in case of UK, for example), they do not always do so. They may refuse to provide the data on the grounds of cost or breach of data protection, or may not have the requested data.

To preview the results, we do not find that observable characteristics of the local area or the police force determine whether the request for provision of hate crime data was not fulfilled at all or in part, nor the speed at which it was responded to. Similarly, university characteristics such as size, age composition and salary of staff, total income, ranking or Research Excellence Framework (REF) submission statistics, do not predict the likelihood of response to the request and provision of data by UK universities. The Research Excellence Framework (REF) is a system for assessing the quality of research in the UK higher education institutions. The outcomes of REF evaluation play a crucial role in determining a university’s future research funding and its reputation (see [12]). This suggests that the data collected from an FOI request are broadly representative of the institutions from which they were requested and that results obtained from analysis using them should be externally valid.

In the next section, we explain the FOI law in the UK and illustrate how to make an FOI request (in the UK). Section III documents the determinants of the two requests we made, Section IV provides potential constraints when making FOI requests, while Section V concludes the paper.

## How to make an FOI request

The Freedom of Information Act 2000 came into force on January 1^st^ 2005 in the UK. It provides access to information held by public authorities by making them publish information about their actions and by allowing the public to request information from them. The UK “enacted FOI within the context of an information revolution that has made government more open and information easier to use, store, access, and distribute” [10]. Also, there was a strong government commitment and political will to implement an effective FOI regime. The UK has an exceptionally wide coverage, with an estimated 100,000 public bodies being subject to the Act all at once: central and local government, the National Health Service (NHS), maintained schools and other educational institutions, armed forces, police, and other public bodies and offices. Together with New Zealand, UK FOI regime fares best, given its openness and high level of political and official support [10].

FOI requests are free, and requests must be made in writing. Authorities typically have a contact email address or an online application form through which the requests can be made. These can be found on dedicated FOI webpages as part of the authority’s website. While it is not necessary to state that the request is made under the FOI act, doing so helps avoiding any confusion and sets the clock ticking. Requests must be acknowledged (although, in practice, not all institutions actually acknowledge the receipt of the request, despite the legal obligation to do so) and answered within 20 working days. If clarification is required, the clock is re-set; the countdown begins again from the day clarification is received.

Before making a request, it is important to explore whether the required data can be found elsewhere because, in such a case, the institution in question is likely to refuse provision of the data and instead direct the researcher to the relevant data source. Furthermore, the data of interest to researchers may have been previously requested. A good starting point is the website www.data.gov.uk which contains datasets from all central government departments, local authorities and various public sector bodies. Many organisations also publish the responses to previous FOI requests in their disclosure logs. Therefore it is useful to search through these already existing sources of data prior to making a new FOI request.

## Determinants of response to the two FOI requests

In this section we examine the determinants of response to the two FOI requests that we have undertaken. When using the data obtained by the FOI requests, a researcher may worry that authorities, which replied to an FOI request, might systematically be different from those, which did not reply; therefore, selection bias may be a concern. This exercise is intended to establish whether any particular characteristics make an authority more likely to respond.

### A request for hate crime data from the UK police forces

#### Data collection

On March 2^nd^ 2017 we contacted 47 police forces in the UK with an identical request (the full text of the request can be found in the supplementary material) to provide monthly statistics on the reported number of hate crimes by type of crime, by ethnicity and by nationality of the victim covering the period from January 2011 to February 2017. The requests were randomly allocated between the three authors with two exceptions. The first exception was Essex police, which was contacted as a pilot area to determine the feasibility of the request. The second exception was Avon and Somerset police; the police force was contacted to determine whether being contacted by an academic based locally might increase the likelihood of a positive response. The characteristics of the response across areas are provided in Table 1. Specifically, the response rate was 98%, with one police force not replying. The request was completed on average within 20 days, the exact time within which institutions are obliged to respond. In 28% of cases the request was completed late, usually due to the need to follow up with clarification. The police forces responded in three ways—by refusing to share any information (11%) due to cost or data unsuitability, by providing some data and by providing all requested data. When only partial information was sent back, it was typically justified by the lack of data or the cost of providing additional data being greater than the cost limits set out in Section IV. Among the successful responses, some of the data provided were not in accordance with the requested format (e.g., providing quarterly or annual instead of monthly data).

**Table 1:** Summary statistics- FOI request to police forces.

**Panel A. Descriptives**			**Panel B. Justification provided**	*Number of police forces*	
Number of police forces	47		*Reasons for refusal to provide data overall*		
Request response rate	98%		Cost	5	
Follow up / clarification required	28%		Not appropriate for this research	1	
Late request completion	28%				
*average completion time (working days)*	*19*.*8*		*Reasons not all data provided*		
Refused data provision	11%		Information not held	5	
Provided data of some kind	89%		Cost	2	
*all requested data*	*44*.*7%*				
*most (or all) requested data*	*78*.*7%*		*Reasons data provided not suitable*		
*unsuitable data*	*10*.*3%*		Wrong information (e.g. annual or quarterly)	3	
			Police force not territorial	2	
**Panel C. Police force characteristics**			**Panel D. Local area characteristics**		
	mean	st.dev.		mean	st.dev.
Total police force	6111.96	12600.76	Disposable income per head in 2016	18640.83	2573.63
Total administrative staff	83.54	93.77	Unemployment rate (16–64 year olds) in 2016	4.49	1.07
Administrative staff (per 100 police force)	2.02	0.81			
Administrative staff (per 100 population)	0.02	0.06	% population working age	63.29	2.62
Number of non-white employees (per 100 police force)	1.50	1.63	% population UK born	88.37	14.97
			% population white	91.63	9.08
Funding in £ (per 10000 population)	2771296	8446972			
			% population Christian	62.78	6.88
Total offences (per 100 police force)	2210.78	643.65	% population no religion	25.19	5.19
Reported hate crimes, 2014–2015 (per 100 population)	0.101	0.11			
Reported racist incidents, 2014–2015 (per 100 population)	0.112	0.102	Migration flow (per 100 population)	0.32	0.26

*Note*: Data come from the following sources: police force characteristics and crime statistics—Home Office; local area demographic characteristics—2011 Census; labour market characteristics—ONS. Information about the police forces refers to year 2015/2016, the most recent data available. The crime statistics capture year 2014/2015. The local area demographics reflects the situation at the time of the 2011 Census. The reference year for labour market characteristics is 2015.

#### Analysis

We want to investigate whether these differential responses were determined by either characteristics and circumstances of the police force or of the area it was covering. We examine two outcomes–i) whether the police force provided any data (*sensu lato*) and ii) whether the provided data were what we requested (e.g., monthly and not quarterly or annual) (*sensu stricto*). We have coded the Right Data such that it equals to one if data were suitable to use in the analysis, and zero otherwise. The latter corresponds to “unsuitable data” in Table 1 (10.3% out of 89% who provided data of some kind). Data were deemed unsuitable for the analysis if they were (a) of annual frequency; (b) of quarterly frequency; (c) entirely different from what we have asked for (e.g., no counts, no types of hate crimes, etc). Overall, Right Data is equal to one for 35 police forces and zero for the remaining 10 police forces, thus providing sufficient variation to make a prediction.

We estimate a probit model of the following form:

$$P({Data}_{i}=1|\;x)=\Phi (\beta_{0}+{PF}_{i}^{'}\gamma +{LA}_{i}^{'}\delta +{LL}_{i}^{'}\theta )$$

where *Data* indicates one of the two responses set out above. Among the determinants of the FOI request we consider police force *(PF)* characteristics including total size, overall funding and staff available to process the requests. Forces with more funding might be better placed to deal with the FOI requests and may be less inclined to divert funds from administrative staff towards more front-line policing. The likelihood of the request being completed and being completed on time should increase with the size of the administrative team. We also examine local area characteristics *(LA)* such as the demographic and ethnic make-up of the area and the proportion of hate crime offences in the period prior to when the request was made. It could be the case that more diverse areas have experienced greater exposure to hate crime and as such have already implemented better data collection practices making it easier to access the data. Finally, we examine the local labour market *(LL)* conditions of the area.

Table 2 presents the results of this analysis with two different dependent variables—whether any data were provided and whether the right data were provided. We present the marginal effects from a set of probit regressions. All regressions include the variables with coefficients reported in the table as well as two dummy variables indicating the contact person on the research team responsible for the request. In columns 1 and 6 we only include the police force characteristics—size per 100 population, number of admin staff per 100 population and funding of the police force per 10000 population. The regressions are run on a sample of 45 police forces, as two police forces (British Transport Police and Doverport) are non-territorial. Hence, there are no local area characteristics available for them. We find no relationship between provision of right data and the police force characteristics. Next, we examine whether the incidence of hate crimes is correlated with the response to the FOI request (columns 2 and 7). We find no relationship. We have also used a more general measure of crime (i.e., total crimes committed per 100 population) instead of the hate crime variable. The regression results remain unchanged. Then we consider the demographic characteristics of the area (columns 3 and 8) and find correlations between provision of any data and the share of population who are white (+), the share of population who are of non-Christian religion (+) and the share of population of working age (–). There is no relationship between these characteristics and provision of right data. Lastly, we consider the role played by the local labour market characteristics (columns 4 and 9) and find no relationship with either of the dependent variables. When we include these variables together in a regression (columns 5 and 10), all coefficients become statistically insignificant. As can be seen from pseudo-R^2^ reported in Table 2, inclusion of various police force characteristics increases the explanatory power of the model, as expected. Overall, we conclude that the above characteristics do not determine the responsiveness of police forces to our FOI request.

**Table 2:** The determinants of the response to an FOI request of all police forces in the UK.

	(1)	(2)	(3)	(4)	(5)	(6)	(7)	(8)	(9)	(10)
Dependent Variable.	Any Data					Right Data				
Size of police force	0.629				0.981	1.209				2.520
(per 100 of the population)	(0.705)				(2.282)	(0.928)				(2.515)
Size of admin team	-22.309				-10.540	-36.396*				-74.989
(per 100 of the population)	(16.233)				(44.503)	(22.095)				(63.694)
Total funding	0.000				-0.000	-0.000				-0.000
(£ per 10000 population)	(0.000)				(0.000)	(0.000)				(0.000)
***Crime***										
Hate crimes		-0.486			10.255		-0.567			0.804
(per 100 population)		(0.416)			(19.099)		(0.508)			(2.980)
***Local population characteristics***										
% population working age			-0.079**		-0.141			-0.045		0.034
			(0.031)		(0.215)			(0.043)		(0.062)
% population white			0.085**		0.37			0.049		0.215
			(0.035)		(0.547)			(0.043)		(0.202)
% population non-UK born			-0.007		0.005			0.014		0.050
			(0.016)		(0.040)			(0.026)		(0.063)
% population of non-Christian religion			0.175***		0.703			0.064		0.249
			(0.062)		(1.049)			(0.066)		(0.234)
***Local labour market***										
disposable income per head				-0.000	-0.000				-0.000	-0.000
				(0.000)	(0.000)				(0.000)	(0.000)
unemployment level				-0.000	0.000				-0.000	0.000
				(0.000)	(0.000)				(0.000)	(0.000)
Pseudo-R2	0.194	0.072	0.235	0.042	0.563	0.153	0.040	0.057	0.022	0.342
Observations	43	43	45	45	42	43	43	45	45	42

*Notes*: The dependent variable is a dummy equal to 1 if any requested data / right data were provided. Reported coefficients are marginal effects from probit regressions. Regressions are run on a sample of 45 police forces, as two police forces (British Transport Police and Doverport) are non-territorial. All regressions include two dummy variables indicating the contact person on the research team responsible for the request. Robust standard errors in parentheses.

*, ** and *** respectively denote statistical significance at the 10, 5 and 1% level.

Data sources: FOI requests, 2011 Census, Home Office and ONS.

#### Robustness checks

In the supplementary material we also provide results (S2 Appendix Table A) of the regressions using two alternative measures: i) whether the police force provided all requested data (i.e., monthly number of hate crimes by type, by ethnicity of victim and by nationality of victim), and ii) whether the request was completed late (i.e., after the 20 working day target). No characteristics we study seem to determine late provision of data. We find positive and marginally significant correlations between the local population characteristics and provision of all requested data. As an additional check (S2 Appendix Table B), we have also dropped two London-based police forces (Metropolitan and City) from the main regressions. This is because due to the London allowance wages paid to administrative staff, processing the requests in London increases the cost of complying. The regression results considering provision of any data remain unaltered. In regressions considering provision of right data, when all controls are included together, the coefficients on some of the local population characteristics become significant but only at 10% level.

Furthermore, due to the potential issue that many police forces provided data of some kind and as such there is little variation in the dependent variable, we estimate a version of the logistic model that takes into account such ‘rare’ events. Specifically, we fit a logistic model by penalized maximum likelihood regression set out by [13] and implemented in Stata by [14]. The results, in the supplementary S2 Appendix (Table C), are qualitatively and quantitatively similar to those obtained using a probit model.

While we have chosen variables which we thought, a priori, were most likely to be determinants of receiving the data it could well be the case that other variables may be more important. To address this issue, we have run a series of alternative specifications using alternative variables to the ones we chose. In Tables D and E of the S2 Appendix, where the dependent variables are “Any Data” and “Right Data” respectively, we have re-estimated the full model, as set out in Table 2 columns 5 and 10, and have in turn replaced each variable (with the exception of those that capture local labour market characteristics, and the proportion of working age population, due to lack of obvious alternatives) with at least one alternative. For example, we have replaced the size of the police force with the size of the police force who are BME (Black and Minority Ethnic), we have replaced hate crimes with violent crimes, and we have made various replacements of ethnicity, country of birth and religionvariables. In total, we have estimated nine different specifications that have used thirteen different variables. Of the 362 parameters estimated, as shown in S2 Appendix Table D, none are statistically significant. In S2 Appendix Table E we find just 5 coefficients which are statistically significant at the 10% level and one at the 5% level. In summary, this additional analysis does not alter our conclusions.

### A request for data on maternity leave uptake at the UK universities

#### Data collection

On August 24^th^ 2018 we contacted 162 universities in the UK with an FOI request. We chose higher education providers registered with the Higher Education Standard Agency (HESA) (https://www.hesa.ac.uk/support/providers). We excluded those which operate under an umbrella of another institution. For example, the Hull York Medical School is a joint venture between the University of York and Hull University. As such the respective universities include information about the Medical School staff in their statistics. The exact wording of the request can be found in the supplementary material. The request asked for individual level data for years 2010–2017 about employees who took maternity leave, including their age, university department they have worked at, pay grade, the number of weeks of leave taken and whether they have taken any shared parental leave with their partners. Furthermore, we also requested the following university and department level statistics for the same period: total number of staff employed and by gender; overall number of professors and split by gender; total number of employees who took maternity or shared parental leave. The same person sent all requests. The response rate and the type of responses provided can be found in Table 3. The overall response rate was high with 78% of contacted universities acknowledging the receipt of the request. The response rate was lower than in the case of police forces discussed above and it is perhaps surprising given the legal duty to respond. This could be due to there not being a credible threat of punishment. The Act can be breached in three ways: 1) fail to respond adequately to a request; 2) fail to adopt the model publication scheme, or do not publish the correct information or 3) deliberately destroy, hide or alter requested information. The third breach is a criminal act whereas the first two are unlawful. The Information Commissioner’s Office (ICO) cannot impose fines if a body fails to comply with the Act, nor can they require the organisation to provide compensation to anyone for breaches of the Act. For further details see: https://ico.org.uk/for-organisations/guide-to-freedom-of-information/complaints/. Of those who responded, 91% provided some data—either as requested or averages (due to concerns about breach of data protection). Only 9% of respondents refused provision of data. In most cases this was due to the data protection concerns; one university argued that they lacked capacity to provide the data and one argued that due to new HR system being introduced, the requested data could not be retrieved.

**Table 3:** Summary statistics—Universities.

**Panel A. Response rate and reasons for not providing data**		
Number of universities	162	
Request response rate	78%	
*Among respondents*:		
Refused data provision	9%	
Provided data of some kind	91%	
*all requested data*	65.50%	
*partial data*	25.50%	
*Reasons for refusal*	*# of universities*	
data protection	8	
new HR system, data unavailable	1	
capacity	1	
**Panel B. Characteristics of universities**		
	Mean	S.D.
Total number of staff employed	2575.09	2532.11
Proportion of staff aged 35 or under	0.28	0.06
Admin staff as proportion of all staff	0.51	0.11
Average academic salary (in thousands £)	49.79	10.47
Average professional services salary (in thousands £)	31.60	3.67
Income in 2016/2017 per member of staff (in thousands £)	83.07	41.20
Rank in the league tables	66.11	37.92
Total number of staff submitted to REF	339.43	449.72
Number of REF 4* publications	20.00	12.41
Number of REF 3* publications	40.39	10.45

*Note*: Data sources: FOI request, HESA data on income and staff composition in 2016/17, The Complete University Guide (league tables), REF website (REF submission statistics).

#### Analysis

As explained earlier, we would like to ensure that the heterogeneity in responses from the higher education institutions is not driven by the systematic differences between them; if this were the case, the external validity of the data would be compromised. We examine two outcomes—i) whether the university responded to the request and ii) whether the requested data were provided. The choice of first outcome was dictated by the fact that, unlike in case of police forces in the UK, many HE institutions simply did not respond to the request. Given the nature of the FOI request one could argue that the universities may refuse to provide data if they lack resources to comply with the request (i.e., have small administrative teams), have small proportion of young and/or female employees who are likely to take maternity leave (as then identification of such individuals from the data is more likely) or perhaps do not have attractive maternity pay policies and thus their employees take short leaves.

Specifically, we estimate a probit model of the following form:

$$P({Data}_{i}=1|\;x)=\Phi (\beta_{0}+{UNI}_{i}^{'}\gamma +{RANK}_{i}^{'}\delta +{REF}_{i}^{'}\theta )$$

where *Data* is an outcome variable as set out above. We consider the following characteristics as determinants of the response and data provision. First, we consider general university *(UNI)* characteristics. These include the number of staff employed, percentage of staff under the age of 35, administrative staff as proportion of all staff, average salaries of academic and administrative staff, institution’s income per member of staff. It might be the case that the bigger the institution, the more likely they are to comply given that they may have less of an issue with the data protection, given a small chance of revealing information about individuals due to publishing small data samples. This might also be the case for those institutions with younger academics. Furthermore, they may have better capacity to process the request.

Second, we consider measures that could signal the quality of the institution. These include league table data (i.e., the university rank) *(RANK)* and information from the Research Excellence Framework 2014 *(REF)* submission, which includes total number of staff submitted to REF, a number of 4-star and a number of 3-star publications (according to the Association of Business Schools (ABS) academic journal guide). We control for the reputation of a given university as we expect more reputable institutions to be more likely to comply with the FOI requests, because they often have dedicated teams who handle all FOI requests.

Results are presented in Table 4. We present the marginal effects from a set of probit regressions. In columns (1) and (5) we include the general university characteristics—the total number of employees, proportion of staff under 35 years of age, administrative staff as proportion of all staff, average salaries and income per member of staff. We find that there is no statistically significant relationship between most of the variables and response rate as well as data provision. There are two exceptions here: first, we find a positive significant relationship between the average academic salary at an institution and the likelihood of responding to the request. Second, institutions with a higher proportion of staff under the age of 35 are also more likely to respond. The first relationship could be indicative of the fact that more generous employers (who also are likely to have better maternity leave provisions) are more willing to share the requested information. The second observation may suggest that institutions which experience higher levels of maternity leave uptake may be more willing to share the data—perhaps because of lesser concerns related to data protection, thanks to the size of the cohort taking leave. Nonetheless, both relationships become insignificant upon inclusion of further controls on REF performance and League Tables ranking of the university.

**Table 4:** The effect of university characteristics on response to the FOI request.

Dependent variable	Responded				Provided requested data			
	(1)	(2)	(3)	(4)	(5)	(6)	(7)	(8)
**University characteristics**								
Total number of staff employed	-0.000			-0.000	-0.000			0.000
	(0.000)			(0.000)	(0.000)			(0.000)
Percentage of staff under 35 years old	1.040*			1.545	0.861			0.951
	(0.567)			(0.963)	(0.643)			(1.037)
Admin staff as proportion of all staff	-0.420			0.407	-0.094			0.694
	(0.410)			(0.536)	(0.465)			(0.602)
Average academic salary (in thousands £)	0.018**			0.011	0.010			0.007
	(0.009)			(0.012)	(0.010)			(0.013)
Average salary of admin staff (in thousands £)	-0.014			0.012	-0.003			0.021
	(0.013)			(0.017)	(0.015)			(0.020)
Income per member of staff (in thousands £)	0.001			0.002	0.001			0.000
	(0.001)			(0.002)	(0.001)			(0.003)
**League Tables rank**		-0.000		0.001		-0.000		0.000
		(0.001)		(0.002)		(0.001)		(0.002)
**REF scores**								
Total staff submitted to REF			-0.000	0.000			-0.000	-0.000
			(0.000)	(0.000)			(0.000)	(0.000)
REF submissions ranked 4*			-0.000	-0.002			0.001	0.007
			(0.004)	(0.008)			(0.004)	(0.009)
REF submissions ranked 3*			0.002	0.003			-0.000	-0.006
			(0.003)	(0.005)			(0.004)	(0.006)
Observations	155	131	151	127	155	131	151	127
**Pseudo-R2**^**2**^	0.060	0.00067	0.0061	0.069	0.025	0.00022	0.0014	0.044

*Note*: Robust standard errors in parentheses.

*** p<0.01,

** p<0.05,

* p<0.1

The reported coefficients are marginal effects from probit regressions. Data sources: FOI request, HESA data on income and staff composition in 2016/17, The Complete University Guide (league tables), REF website (REF submission statistics).

We then also examine whether the ranking in the university league tables plays a role for the analysed outcomes (columns (2) and (6)). We find no meaningful relationships. Subsequently we investigate correlations between the response to the request and data provision and the REF outcomes of the institutions (columns (3) and (7))—none of the REF characteristics determine the outcomes in question. Lastly, we include all the characteristics in the final regression and, as before, uncover no statistically significant relationships. As can be seen from pseudo-R^2^ reported in Table 4, inclusion of various university characteristics increases the explanatory power of the model, as expected, but overall the control variables explain relatively small proportion of variation in the dependent variable. Therefore, we conclude that the observable characteristics of HE institutions do not determine whether and in what way they respond to the FOI request.

#### Robustness checks

Due to potential concerns over the lack of variability in the dependent variables, we have also fitted a logistic model by penalized maximum likelihood regression using the same regression specification as above. The results are qualitatively and quantitatively similar to those estimated using a probit model and can be found in the supplementary material (S2 Appendix Table F).

In addition, we have also used alternative variables to those used in the main analysis to verify whether the choice of control variables may have driven the reported lack of correlations. For example, given the gendered nature of the FOI request, one may be concerned that it is not the university characteristics as a whole but rather female-specific characteristics which may determine an institution’s response to such a request. Therefore, in S2 Appendix Table G in the supplementary material we have replaced some of the university characteristics with gender-focused alternatives, where possible. For example, we have used the proportion of female staff rather than total staff numbers and we have used the female to male wage ratios, separately for professional and academic staff, instead of average wages. These results are similar to those found in the main analysis and do not alter our conclusions.

## FOI requests—Potential constraints

There are several constraints, which the researcher faces when making an FOI request. First, knowing whether the data required is available is sometimes difficult to ascertain. As described above, a priori search of the disclosure logs or the central government data collection website (data.gov.uk) can help narrow this down. However, the answer may still not be obvious. Therefore, in order to improve the success of a request, particularly when contacting multiple authorities with the same request (for example, in our FOI requests we contacted every police force and every higher education institution in the country), we recommend choosing one or two authorities to pilot the request. This piloting would allow one to see whether the data are collected and held by the authority (department). In the case of requests to only one department an informal email asking about data availability might suffice.

The second major constraint is cost. A request can be refused if the cost of releasing the data exceeds £600 for central government, Parliament and the armed forces, and £450 for other public authorities. This threshold is based on a standard rate of £25 per person per hour, meaning that a request can be refused if more than 18 hours are needed to complete it. The piloting will also allow the researcher to gauge whether the request is within the cost limits of the FOI Act.

The third major constraint is the protection and privacy of individual data. According to Part II, Section 40 of the FOI legislation a public body may refuse a request if personal information is being requested. All exemptions can be found in Part II, Sections 21–44 of the Freedom of Information Act 2000; many examples are discussed here: https://www.whatdotheyknow.com/. A further complication is a recent roll out of the General Data Protection Regulation (GDPR), which addresses protection and privacy of individual data within the European Union (EU) and the European Economic Area (EEA), as well as the export of personal data outside of EU and EEA areas. Taking these two jointly, the FOI data request might be refused on the ground that it leads to identification of particular individuals. For example, in our request to Higher Education institutions in the UK, we asked for provision of anonymised, individual level data regarding uptake of maternity leave, seniority level and age of an employee. In case of small departments within universities, the request may have permitted identification of an individual despite data being anonymised, because very few individuals took maternity leave in a given period. Therefore, these institutions refused to provide the data.

The format of the data poses the fourth constraint. Obtaining data from a number of public authorities involves sending multiple requests, and various institutions collect and catalogue the data in different ways. This heterogeneity of the data collection process makes it unlikely that requested data are provided in the same format by all institutions, which then increases the cost of data processing by a researcher. Therefore, we suggest providing the authority with an example of the data format that is required, i.e., an empty excel sheet with an exemplary data format.

If an FOI request is denied, the authority will reply setting out the reasons for refusal. If the provided reasons are not satisfactory, one can in first instance ask for an internal review of the decision (by writing back to the authority refusing the request), and subsequently appeal to the Information Commissioner’s Office (ICO). The ICO is an independent regulatory office that deals with Data Protection Act 1998, Privacy and Electronic Communications (EC Directive) Regulations 2003, the Freedom of Information Act 2000, and the Environmental Information Regulations 2004. At each stage, the initial request and responses will be reviewed. However, appeals happen infrequently and the process is likely to be lengthy. A third of complaints took over three months to resolve in 2015/16.

## Conclusions

Questions like what is the relationship between Brexit and the rise of race and religious hate crime or what is the uptake of the shared parental leave and by whom stand high on the research and policy agenda in the UK. Despite their high visibility and importance for policy-making, these topics are still heavily under-researched. This is largely due to the lack of available quantitative data, which would permit rigorous empirical (econometric) analysis. We have put forward what we believe to be an underused (by economists and other social scientists) method of collecting a potentially unique dataset—a Freedom of Information (FOI) request. We briefly describe how to make a request with a focus on researchers who in the main will be requesting the data. Our two main tips to improve the success of requests are i) to pilot the request and ii) provide the authority with an example of the required data format. Finally, we examine the drivers of the two requests we recently made—one to all police forces in the UK and one to all Higher Education institutions in the UK. We find that the observable characteristics are not significantly correlated with the probability that a request was satisfied, suggesting that the data from these FOI requests are broadly representative of the institutions from which they were requested. We further recommend that researchers using FOI to gather data compare responders and non-responders to examine if replies are driven (at least) by observable characteristics.

## Supporting information

S1 Appendix Text of the FOI request 1. (DOCX)

S2 Appendix Determinants of the FOI request. (DOCX)
