# Supplementary material for: Freedom of Information (FOI) as a data collection tool for social scientists
Source: PLoS One. 2020 Feb 21;15(2):e0228392. doi: 10.1371/journal.pone.0228392 (PMC7034795; doi:10.1371/journal.pone.0228392)
Supplement: S1 Appendix — (DOCX) [file pone.0228392.s001.docx]

**S1 Appendix. Text of the FOI request 1**

Below is the text of the FOI request we sent to all police forces in the UK (as referred to on in the text*):*

*Dear Sir/Madam,*

*Under the Freedom of Information Act I would like to request the following data:*

*1) The number of hate crimes each month from January 2011 to February 2017 broken down by the type of hate crime.*

*2) The number of hate crimes each month from January 2011 to February 2017 broken down by the country of origin of the victim.*

*3) The number of hate crimes each month from January 2011 to February 2017 broken down by the ethnicity of the victim.*

*Please could this be provided in an excel format. I have attached an example excel workbook for your convenience. Any other electronic format would also be welcomed.*

*Best wishes,*

*XXX*
