# Supplementary material for: Freedom of Information (FOI) as a data collection tool for social scientists
Source: PLoS One. 2020 Feb 21;15(2):e0228392. doi: 10.1371/journal.pone.0228392 (PMC7034795; doi:10.1371/journal.pone.0228392)
Supplement: S2 Appendix — (DOCX) [file pone.0228392.s002.docx]

**S2 Appendix. Determinants of the FOI request**

Below we present additional results of regressions (as referred to in the text) using the following variables as the dependent variable: i) whether the data was provided late, ii) whether all requested data was provided.

**Table A. The determinants of the response to an FOI request to all police forces in the UK**

| **Dependent variable** | **Late** | | | | | **All Data** | | | | |
| --- | --- | --- | --- | --- | --- | --- | --- | --- | --- | --- |
|  | (1) | (2) | (3) | (4) | (5) | (6) | (7) | (8) | (9) | (10) |
| ***Police force characteristics*** |  |  |  |  |  |  |  |  |  |  |
| size of the police force | -1.813* |  |  |  | -0.000 | 1.728 |  |  |  | 1.950 |
| (per 100 population) | (0.975) |  |  |  | (0.000) | (1.111) |  |  |  | (1.515) |
| size of admin team | 35.035 |  |  |  | 55.452 | -9.369 |  |  |  | -0.100 |
| (per 100 population) | (21.968) |  |  |  | (79.134) | (20.759) |  |  |  | (40.052) |
| total funding | 0.000* |  |  |  | -0.000 | -0.000 |  |  |  | -0.000* |
| (per 10000 population) | (0.000) |  |  |  | (0.000) | (0.000) |  |  |  | (0.000) |
| ***Incidence of hate crime*** |  |  |  |  | -0.173 |  |  |  |  | 0.054 |
| # hate crimes |  | 1.416 |  |  | (0.310) |  | -0.374 |  |  | (0.246) |
| (per 100 population) |  | (0.863) |  |  | -0.120 |  | (0.706) |  |  | 0.451 |
| ***Local population characteristics*** |  |  |  |  | (0.239) |  |  |  |  | (0.285) |
| % population working age |  |  | 0.059 |  | 3.973 |  |  | -0.141*** |  | 1.392 |
|  |  |  | (0.047) |  | (6.856) |  |  | (0.054) |  | (3.027) |
| % population white |  |  | -0.084* |  | -0.035 |  |  | 0.119** |  | -0.061 |
|  |  |  | (0.048) |  | (0.076) |  |  | (0.054) |  | (0.082) |
| % population non-UK born |  |  | -0.026 |  | -0.408 |  |  | 0.046 |  | 0.254** |
|  |  |  | (0.030) |  | (0.649) |  |  | (0.033) |  | (0.120) |
| % population of non-Christian religion |  |  | -0.086 |  | -0.113 |  |  | 0.175** |  | 0.108* |
|  |  |  | (0.067) |  | (0.189) |  |  | (0.085) |  | (0.064) |
| ***Local labour market*** |  |  |  |  | -0.480 |  |  |  |  | 0.297* |
| disposable income per head |  |  |  | 0.000* | (0.779) |  |  |  | -0.000 | (0.157) |
|  |  |  |  | (0.000) | 0.000 |  |  |  | (0.000) | -0.000 |
| unemployment level |  |  |  | 0.000 | (0.000) |  |  |  | 0.000 | (0.000) |
|  |  |  |  | (0.000) | 0.000 |  |  |  | (0.000) | 0.000 |
| Observations | 42 | 42 | 45 | 44 | 41 | 43 | 43 | 46 | 45 | 42 |
| Pseudo-R2 | 0.130 | 0.084 | 0.176 | 0.075 | 0.431 | 0.099 | 0.034 | 0.154 | 0.044 | 0.294 |

*Notes*: The dependent variable is a dummy equal to 1 if late/all data requested data were provided. Reported coefficients are marginal effects from probit regressions. Regressions are run on a sample of 45 police forces, as two police forces (British Transport Police and Doverport) are non-territorial. Column 5 replaces size of the police force (per 100 population) with the size of the police force not taking into account population size due to conformability issues of the probit regression. All regressions include two dummy variables indicating the contact person on the research team responsible for the request. Robust standard errors in parentheses. *, ** and *** respectively denote statistical significance at the 10, 5 and 1% level. Data sources: FOI requests, 2011 Census, Home Office and ONS.

**Table B. The determinants of the response to an FOI request of police forces in the UK (excluding City of London and the Metropolitan Police Service)**

| **Dependent variable** | **Any Data** | | | | | **Right Data** | | | | | |
| --- | --- | --- | --- | --- | --- | --- | --- | --- | --- | --- | --- |
|  | (1) | (2) | (3) | (4) | (5) | | (6) | (7) | (8) | (9) | (10) |
| ***Police force characteristics*** |  |  |  |  |  | |  |  |  |  |  |
| size of the police force | 0.523 |  |  |  | 0.008 | | 1.082 |  |  |  | 1.948 |
| (per 100 population) | (0.754) |  |  |  | (0.024) | | (0.896) |  |  |  | (1.116) |
| size of admin team | -10.668 |  |  |  | -0.084 | | -29.252 |  |  |  | -60.351 |
| (per 100 population) | (18.571) |  |  |  | (0.383) | | (24.171) |  |  |  | (30.912) |
| total funding | 0.000 |  |  |  | -0.000 | | 0.000 |  |  |  | -0.000 |
| (per 10000 population) | (0.000) |  |  |  | (0.000) | | (0.000) |  |  |  | (0.000) |
| ***Incidence of hate crime*** |  |  |  |  |  | |  |  |  |  |  |
| # hate crimes |  | 2.389 |  |  | 0.081 | |  | 1.602 |  |  | 0.867 |
| (per 100 population) |  | (1.482) |  |  | (0.201) | |  | (1.439) |  |  | (2.664) |
| ***Local population characteristics*** |  |  |  |  |  | |  |  |  |  |  |
| % population working age |  |  | -0.076* |  | -0.001 | |  |  | -0.057 |  | 0.026 |
|  |  |  | (0.034) |  | (0.003) | |  |  | (0.046) |  | (0.038) |
| % population white |  |  | 0.078* |  | 0.003 | |  |  | 0.058 |  | 0.206 |
|  |  |  | (0.035) |  | (0.008) | |  |  | (0.044) |  | (0.113) |
| % population non-UK born |  |  | -0.012 |  | 0.000 | |  |  | 0.004 |  | 0.043 |
|  |  |  | (0.014) |  | (0.000) | |  |  | (0.027) |  | (0.040) |
| % population of non-Christian religion |  |  | 0.159* |  | 0.006 | |  |  | 0.082 |  | 0.246 |
|  |  |  | (0.063) |  | (0.015) | |  |  | (0.066) |  | (0.142) |
| ***Local labour market*** |  |  |  |  |  | |  |  |  |  |  |
| disposable income per head |  |  |  | -0.000 | -0.000 | |  |  |  | -0.000 | -0.000 |
|  |  |  |  | (0.000) | (0.000) | |  |  |  | (0.000) | (0.000) |
| unemployment level |  |  |  | -0.000 | 0.000 | |  |  |  | -0.000 | 0.000 |
|  |  |  |  | (0.000) | (0.000) | |  |  |  | (0.000) | (0.000) |
| Observations | 41 | 41 | 44 | 43 | 40 | | 41 | 41 | 44 | 43 | 40 |
| Pseudo-R2 | 0.133 | 0.161 | 0.277 | 0.075 | 0.511 | | 0.094 | 0.058 | 0.075 | 0.055 | 0.302 |

*Notes*: The dependent variable is a dummy equal to 1 if any/right data requested data were provided. Reported coefficients are marginal effects from probit regressions. Regressions are run on a sample of 45 police forces, as two police forces (British Transport Police and Doverport) are non-territorial. All regressions include two dummy variables indicating the contact person on the research team responsible for the request. Robust standard errors in parentheses. *, ** and *** respectively denote statistical significance at the 10, 5 and 1% level. Data sources: FOI requests, 2011 Census, Home Office and ONS

**Table C. The determinants of the response to an FOI request of all police forces in the UK (Estimation of logistic model by penalized maximum likelihood regression)**

| **Dependent variable** | **Any Data** | | | | | **Right_Data** | | | | |
| --- | --- | --- | --- | --- | --- | --- | --- | --- | --- | --- |
|  | (1) | (2) | (3) | (4) | (5) | (6) | (7) | (8) | (9) | (10) |
| ***Police force characteristics*** | | | | | | | | | | |
| size of the police force | 1.681 |  |  |  | 2.136 | 4.291 |  |  |  | 5.365 |
| (per 100 population) | (6.262) |  |  |  | (6.625) | (6.158) |  |  |  | (6.803) |
| size of admin team | -103.167 |  |  |  | -104.934 | -145.001 |  |  |  | -175.916 |
| (per 100 population) | (123.910) |  |  |  | (163.770) | (129.772) |  |  |  | (158.433) |
| total funding | 0.000 |  |  |  | 0.000 | 0.000 |  |  |  | 0.000 |
| (per 10000 population.) | (0.000) |  |  |  | (0.000) | (0.000) |  |  |  | (0.000) |
| ***Incidence of hate crime*** | | | | | | | | | | |
| number of hate crimes |  | -3.131 |  |  | -2.245 |  | -2.557 |  |  | -4.890 |
| (per 100 population) |  | (2.455) |  |  | (15.663) |  | (2.396) |  |  | (17.587) |
| ***Local population characteristics*** | | | | | | | | | | |
| % population working age |  |  | -0.561* |  | -0.056 |  |  | -0.184 |  | 0.120 |
|  |  |  | (0.324) |  | (0.389) |  |  | (0.204) |  | (0.299) |
| % population white |  |  | 0.624* |  | 0.443 |  |  | 0.220 |  | 0.313 |
|  |  |  | (0.363) |  | (0.377) |  |  | (0.213) |  | (0.401) |
| % population non-UK born |  |  | -0.035 |  | -0.042 |  |  | 0.071 |  | 0.076 |
|  |  |  | (0.180) |  | (0.223) |  |  | (0.134) |  | (0.213) |
| % population of non-Christian religion |  |  | 1.250* |  | 0.813 |  |  | 0.271 |  | 0.372 |
|  |  |  | (0.727) |  | (0.704) |  |  | (0.302) |  | (0.606) |
| ***Local labour market*** | | | | | | | | | | |
| disposable income per head |  |  |  | -0.000 | 0.000 |  |  |  | -0.000 | 0.000 |
|  |  |  |  | (0.000) | (0.000) |  |  |  | (0.000) | (0.000) |
| unemployment level |  |  |  | -0.000 | 0.000 |  |  |  | -0.000 | 0.000 |
|  |  |  |  | (0.000) | (0.000) |  |  |  | (0.000) | (0.000) |
| Observations | 43 | 43 | 45 | 45 | 42 | 43 | 43 | 45 | 45 | 42 |
| Chi-squared | 0.459 | 0.446 | 0.503 | 0.792 | 0.875 | 0.542 | 0.609 | 0.869 | 0.903 | 0.954 |

*Notes*: The dependent variable is a dummy equal to 1 if any/right data requested data were provided. Reported coefficients are marginal effects from a Penalized maximum likelihood logistic regression. Regressions are run on a sample of 45 police forces, as two police forces (British Transport Police and Doverport) are non-territorial. All regressions include two dummy variables indicating the contact person on the research team responsible for the request. Robust standard errors in parentheses. *, ** and *** respectively denote statistical significance at the 10, 5 and 1% level. Data sources: FOI requests, 2011 Census, Home Office and ONS

**Table D. The (alternative) determinants of the response to an FOI request of all police forces in the UK**

| Dependent variable | Any Data | | | | | | | | |
| --- | --- | --- | --- | --- | --- | --- | --- | --- | --- |
|  | (1) | (2) | (3) | (4) | (5) | (6) | (7) | (8) | (9) |
| ***Police force characteristics*** | | | |  |  |  |  |  |  |
| Size of police force | 0.981 |  | 0.980 | 6.092 | 0.403 | 1.728 | 0.617 | 1.345 | 1.418 |
| (per 100 population) | (2.282) |  | (2.186) | (8.559) | (2.135) | (2.614) | (1.746) | (2.410) | (3.480) |
| Size of admin team | -10.540 | 0.000 |  | -100.662 | -14.866 | -66.424 | -8.609 | -23.518 | -12.967 |
| (per 100 population) | (44.503) | (0.295) |  | (164.089) | (82.336) | (75.376) | (27.821) | (57.483) | (43.897) |
| Total funding | -0.000 | -0.000 | -0.000 |  | 0.000 | -0.000 | -0.000 | -0.000 | -0.000 |
| (£ per 10000 population) | (0.000) | (0.000) | (0.000) |  | (0.000) | (0.000) | (0.000) | (0.000) | (0.000) |
| Size of BME police force |  | 0.320 |  |  |  |  |  |  |  |
| (per 100 population) |  | (0.709) |  |  |  |  |  |  |  |
| Size of police staff admin team |  |  | -11.222 |  |  |  |  |  |  |
| (per 100 population) |  |  | (42.558) |  |  |  |  |  |  |
| Core funding |  |  |  | -0.000 |  |  |  |  |  |
| (£ per 10000 population) |  |  |  | (0.001) |  |  |  |  |  |
| ***Crime*** |  |  |  |  |  |  |  |  |  |
| Hate crimes | 10.255 | 0.102 | 10.023 | 16.481 |  |  | 1.091 | 12.706 | 12.329 |
| (per 100 population) | (19.099) | (0.230) | (18.407) | (21.196) |  |  | (3.952) | (19.523) | (30.314) |
| Violence with injury crimes |  |  |  |  | -0.099 |  |  |  |  |
| (per 100 population) |  |  |  |  | (0.457) |  |  |  |  |
| Violence without injury crimes |  |  |  |  | -0.009 |  |  |  |  |
| (per 100 population) |  |  |  |  | (0.091) |  |  |  |  |
| Public order offences |  |  |  |  |  | -0.174 |  |  |  |
| (per 100 population) |  |  |  |  |  | (0.589) |  |  |  |
| Robbery |  |  |  |  |  | 3.860 |  |  |  |
| (per 100 population) |  |  |  |  |  | (6.292) |  |  |  |
| ***Local population characteristics*** |  |  |  |  |  |  |  |  |  |
| % population working age | -0.141 | -0.002 | -0.140 | -0.293 | -0.015 | -0.100 | -0.021 | -0.162 | -0.006 |
|  | (0.215) | (0.004) | (0.211) | (0.423) | (0.085) | (0.105) | (0.053) | (0.231) | (0.056) |
| % population white | 0.370 | 0.004 | 0.368 | 0.673 | 0.062 | 0.357 |  | 0.489 | 0.347 |
|  | (0.547) | (0.009) | (0.534) | (0.904) | (0.332) | (0.270) |  | (0.558) | (0.736) |
| % population Asian |  |  |  |  |  |  | 0.017 |  |  |
|  |  |  |  |  |  |  | (0.053) |  |  |
| % population Black |  |  |  |  |  |  | 0.057 |  |  |
|  |  |  |  |  |  |  | (0.133) |  |  |
| % population non-UK born | 0.005 | 0.000 | 0.005 | -0.028 | 0.001 | -0.015 | -0.013 |  | 0.061 |
|  | (0.040) | (0.001) | (0.039) | (0.074) | (0.012) | (0.057) | (0.038) |  | (0.126) |
| % population born Europe (other) |  |  |  |  |  |  |  | -0.124 |  |
|  |  |  |  |  |  |  |  | (0.183) |  |
| % population of non-Christian religion | 0.703 | 0.007 | 0.700 | 1.332 | 0.113 | 0.644 | -0.005 | 0.974 |  |
|  | (1.049) | (0.017) | (1.026) | (1.800) | (0.601) | (0.474) | (0.018) | (1.093) |  |
| % population of Muslim |  |  |  |  |  |  |  |  | 0.650 |
|  |  |  |  |  |  |  |  |  | (1.369) |
| % population of Sikh |  |  |  |  |  |  |  |  | 1.366 |
|  |  |  |  |  |  |  |  |  | (2.872) |
| ***Local labour market*** |  |  |  |  |  |  |  |  |  |
| disposable income per head | 0.703 | 0.007 | 0.700 | 1.332 | 0.113 | 0.644 | -0.005 | -0.000 |  |
|  | (1.049) | (0.017) | (1.026) | (1.800) | (0.601) | (0.474) | (0.018) | (0.000) |  |
| unemployment level | -0.000 | -0.000 | -0.000 | -0.000 | 0.000 | 0.000 | -0.000 | 0.000 | 0.000 |
|  | (0.000) | (0.000) | (0.000) | (0.000) | (0.000) | (0.000) | (0.000) | (0.000) | (0.000) |
| Observations | 42 | 42 | 42 | 42 | 42 | 42 | 42 | 42 | 42 |
| Pseudo-R2 | 0.563 | 0.562 | 0.564 | 0.649 | 0.531 | 0.511 | 0.451 | 0.573 | 0.405 |

*Notes*: The dependent variable is a dummy equal to 1 if any data requested data were provided. Reported coefficients are marginal effects from probit regressions. BME stands for black and minority ethnic. All regressions include two dummy variables indicating the contact person on the research team responsible for the request. Robust standard errors in parentheses. *, ** and *** respectively denote statistical significance at the 10, 5 and 1% level. Data sources: FOI requests, 2011 Census, Home Office and ONS.

**Table E. The (alternative) determinants of the response to an FOI request of all police forces in the UK**

| Dependent variable | Right Data | | | | | | | | |
| --- | --- | --- | --- | --- | --- | --- | --- | --- | --- |
|  | (1) | (2) | (3) | (4) | (5) | (6) | (7) | (8) | (9) |
| ***Police force characteristics*** | | | |  |  |  |  |  |  |
| Size of police force | 2.520 |  | 2.175 | 4.622 | 4.311 | 3.177 | 3.001 | 2.541 | 3.337* |
| (per 100 of the population) | (2.515) |  | (2.679) | (4.796) | (2.917) | (2.487) | (2.366) | (2.315) | (1.734) |
| Size of admin team | -74.989 | -63.394 |  | -101.606 | -129.502* | -101.733 | -68.219 | -78.072 | -87.362** |
| (per 100 of the population) | (63.694) | (43.005) |  | (90.758) | (69.479) | (64.292) | (68.253) | (59.782) | (43.294) |
| Total funding | -0.000 | 0.000 | -0.000 |  | -0.000 | -0.000 | -0.000 | -0.000 | -0.000 |
| (£ per 10000 population) | (0.000) | (0.000) | (0.000) |  | (0.000) | (0.000) | (0.000) | (0.000) | (0.000) |
| Size of BME police force |  | 8.949 |  |  |  |  |  |  |  |
| (per 100 of the population) |  | (31.015) |  |  |  |  |  |  |  |
| Size of police staff admin team |  |  | -65.040 |  |  |  |  |  |  |
| (per 100 of the population) |  |  | (72.293) |  |  |  |  |  |  |
| Core funding |  |  |  | -0.000 |  |  |  |  |  |
| (£ per 10000 population) |  |  |  | (0.000) |  |  |  |  |  |
| ***Crime*** |  |  |  |  |  |  |  |  |  |
| Hate crimes | 0.804 | 1.879 | 0.316 | 2.378 |  |  | 3.122 | 0.655 | 3.886 |
| (per 100 population) | (2.980) | (3.866) | (2.567) | (3.848) |  |  | (4.779) | (3.109) | (4.616) |
| Violence with injury crimes |  |  |  |  | -0.729 |  |  |  |  |
| (per 100 population) |  |  |  |  | (1.094) |  |  |  |  |
| Violence without injury crimes |  |  |  |  | 0.181 |  |  |  |  |
| (per 100 population) |  |  |  |  | (0.424) |  |  |  |  |
| Public order offences |  |  |  |  |  | -0.080 |  |  |  |
| (per 100 population) |  |  |  |  |  | (0.426) |  |  |  |
| Robbery |  |  |  |  |  | -2.245 |  |  |  |
| (per 100 population) |  |  |  |  |  | (5.256) |  |  |  |
| ***Local population characteristics*** |  |  |  |  |  |  |  |  |  |
| % population working age | 0.034 | 0.022 | 0.027 | 0.023 | 0.055 | 0.042 | 0.022 | 0.059 | 0.036 |
|  | (0.063) | (0.068) | (0.056) | (0.046) | (0.074) | (0.066) | (0.057) | (0.076) | (0.061) |
| % population white | 0.215 | 0.280* | 0.192 | 0.255 | 0.325* | 0.264 |  | 0.151 | 0.127* |
|  | (0.202) | (0.151) | (0.232) | (0.236) | (0.186) | (0.169) |  | (0.128) | (0.070) |
| % population Asian |  |  |  |  |  |  | 0.058 |  |  |
|  |  |  |  |  |  |  | (0.094) |  |  |
| % population Black |  |  |  |  |  |  | 0.033 |  |  |
|  |  |  |  |  |  |  | (0.089) |  |  |
| % population non-UK born | 0.050 | 0.085 | 0.042 | 0.060 | 0.053 | 0.066 | 0.001 |  | 0.050 |
|  | (0.063) | (0.063) | (0.063) | (0.062) | (0.064) | (0.071) | (0.051) |  | (0.055) |
| % population born Europe (other) |  |  |  |  |  |  |  | 0.042 |  |
|  |  |  |  |  |  |  |  | (0.088) |  |
| % population born Asia |  |  |  |  |  |  |  | -0.191 |  |
|  |  |  |  |  |  |  |  | (0.279) |  |
| % population of non-Christian religion | 0.249 | 0.298 | 0.225 | 0.298 | 0.400* | 0.323 | -0.099 | 0.274 |  |
|  | (0.234) | (0.190) | (0.272) | (0.279) | (0.225) | (0.207) | (0.096) | (0.265) |  |
| % population of Muslim |  |  |  |  |  |  |  |  | 0.176 |
|  |  |  |  |  |  |  |  |  | (0.126) |
| % population of Sikh |  |  |  |  |  |  |  |  | -0.066 |
|  |  |  |  |  |  |  |  |  | (0.158) |
| ***Local labour market*** |  |  |  |  |  |  |  |  |  |
| disposable income per head | -0.000 | -0.000 | 0.000 | -0.000 | 0.000 | -0.000 | -0.000 | 0.000 | -0.000 |
|  | (0.000) | (0.000) | (0.000) | (0.000) | (0.000) | (0.000) | (0.000) | (0.000) | (0.000) |
| unemployment level | 0.000 | 0.000 | 0.000 | 0.000 | 0.000* | 0.000 | 0.000 | 0.000 | 0.000 |
|  | (0.000) | (0.000) | (0.000) | (0.000) | (0.000) | (0.000) | (0.000) | (0.000) | (0.000) |
| Observations | 42 | 42 | 42 | 42 | 42 | 42 | 42 | 42 | 42 |
| Pseudo-R2 | 0.343 | 0.285 | 0.352 | 0.397 | 0.365 | 0.348 | 0.274 | 0.335 | 0.323 |

*Notes*: The dependent variable is a dummy equal to 1 if the right data requested data were provided. Reported coefficients are marginal effects from probit regressions. BME stands for black and minority ethnic. All regressions include two dummy variables indicating the contact person on the research team responsible for the request. Robust standard errors in parentheses. *, ** and *** respectively denote statistical significance at the 10, 5 and 1% level. Data sources: FOI requests, 2011 Census, Home Office and ONS.

**Table F. The effect of university characteristics on response to the FOI request (Estimation of logistic model by penalized maximum likelihood regression)**

| **Dependent variable** | **Responded** | | | | **Provided requested data** | | | |
| --- | --- | --- | --- | --- | --- | --- | --- | --- |
|  | (1) | (2) | (3) | (4) | (5) | (6) | (7) | (8) |
| **University characteristics** |  |  |  |  |  |  |  |  |
| Total number of staff employed | -0.000 |  |  | -0.000 | -0.000 |  |  | 0.000 |
|  | (0.000) |  |  | (0.000) | (0.000) |  |  | (0.000) |
| Percentage of staff under 35 years old | 6.055* |  |  | 9.599 | 3.927 |  |  | 4.709 |
|  | (3.661) |  |  | (6.130) | (3.242) |  |  | (5.200) |
| Admin staff as proportion of all staff | -2.282 |  |  | 3.160 | -0.631 |  |  | 3.618 |
|  | (2.636) |  |  | (3.567) | (2.251) |  |  | (3.222) |
| Average academic salary (in thousands £) | 0.114* |  |  | 0.070 | 0.005 |  |  | 0.033 |
|  | (0.059) |  |  | (0.090) | (0.025) |  |  | (0.079) |
| Average salary of admin staff (in thousands £) | -0.081 |  |  | 0.076 | 0.007 |  |  | 0.103 |
|  | (0.079) |  |  | (0.115) | (0.068) |  |  | (0.105) |
| Income per member of staff (in thousands £) | 0.003 |  |  | 0.012 | 0.001 |  |  | 0.001 |
|  | (0.005) |  |  | (0.019) | (0.004) |  |  | (0.017) |
| **League Tables rank** |  | -0.002 |  | 0.006 |  | -0.001 |  | 0.000 |
|  |  | (0.006) |  | (0.013) |  | (0.005) |  | (0.012) |
| **REF scores** |  |  |  |  |  |  |  |  |
| Total staff submitted to REF |  |  | -0.000 | 0.000 |  |  | -0.000 | -0.002 |
|  |  |  | (0.000) | (0.002) |  |  | (0.000) | (0.002) |
| REF submissions ranked 4* |  |  | -0.004 | -0.016 |  |  | 0.001 | 0.033 |
|  |  |  | (0.020) | (0.054) |  |  | (0.019) | (0.049) |
| REF submissions ranked 3* |  |  | 0.018 | 0.020 |  |  | 0.002 | -0.027 |
|  |  |  | (0.021) | (0.036) |  |  | (0.020) | (0.034) |
|  |  |  |  |  |  |  |  |  |
| Observations | 155 | 131 | 151 | 127 | 155 | 131 | 151 | 127 |
| Chi-squared | 6.66 | 0.086 | 1.24 | 6.30 | 2.17 | 0.032 | 0.32 | 4.65 |
| p-value | 0.35 | 0.77 | 0.74 | 0.79 | 0.90 | 0.86 | 0.96 | 0.91 |

*Note:* Robust standard errors in parentheses. *** p<0.01, ** p<0.05, * p<0.1 The reported coefficients are marginal effects from a Penalized maximum likelihood logistic regression. Data sources: FOI request, HESA data on income and staff composition in 2016/17, The Complete University Guide (league tables), REF website (REF submission statistics).

**Table G. The effect of (alternative) university characteristics on response to the FOI request**

| Dependent variable | Responded | | |  | Provided Requested Data | | |
| --- | --- | --- | --- | --- | --- | --- | --- |
|  | (1) | (2) | (3) |  | (4) | (5) | (6) |
| **University characteristics** |  |  |  |  |  |  |  |
| % Female staff |  | 0.341 | 0.635 |  |  | 0.931 | 0.777 |
|  |  | (0.735) | (1.005) |  |  | (0.814) | (1.132) |
| % Admin staff |  | -0.279 | 0.059 |  |  | -0.334 | 0.067 |
|  |  | (0.405) | (0.478) |  |  | (0.426) | (0.538) |
| Income per member of staff (in thousands £) |  | -0.000 | 0.002 |  |  | 0.000 | 0.001 |
|  |  | (0.001) | (0.003) |  |  | (0.001) | (0.003) |
| female wage as % of male wage, academic | -0.005 | -0.006 | -0.016* |  | -0.007 | -0.008 | -0.014 |
|  | (0.004) | (0.005) | (0.008) |  | (0.005) | (0.006) | (0.009) |
| female wage as % of male wage, non-academic | -0.009 | -0.009 | -0.010 |  | -0.004 | -0.003 | 0.000 |
|  | (0.006) | (0.006) | (0.007) |  | (0.006) | (0.006) | (0.007) |
| Average age, non-academic |  | -0.019 | -0.023 |  |  | -0.007 | -0.009 |
|  |  | (0.015) | (0.020) |  |  | (0.016) | (0.021) |
| Average age, academic |  | -0.008 | -0.012 |  |  | -0.013 | -0.026 |
|  |  | (0.013) | (0.026) |  |  | (0.014) | (0.032) |
| **League Tables rank** |  |  |  |  |  |  |  |
| League Rank |  |  | 0.002 |  |  |  | 0.002 |
|  |  |  | (0.002) |  |  |  | (0.002) |
| **REF scores** |  |  |  |  |  |  |  |
| Total staff submitted to REF |  |  | -0.000 |  |  |  | -0.000 |
|  |  |  | (0.000) |  |  |  | (0.000) |
| REF submissions ranked 4* |  |  | 0.001 |  |  |  | 0.009 |
|  |  |  | (0.008) |  |  |  | (0.009) |
| REF submissions ranked 3* |  |  | 0.004 |  |  |  | -0.003 |
|  |  |  | (0.005) |  |  |  | (0.006) |
| Observations | 154 | 154 | 127 |  | 154 | 154 | 127 |
| pseudo R-squared | 0.022 | 0.042 | 0.075 |  | 0.012 | 0.027 | 0.043 |

*Note:* Robust standard errors in parentheses. *** p<0.01, ** p<0.05, * p<0.1 The reported coefficients are marginal effects from probit regressions. Data sources: FOI request, HESA data on income and staff composition in 2016/17, The Complete University Guide (league tables), REF website (REF submission statistics).
